# *GPC1* Regulated by *miR-96-5p*, Rather than *miR-182-5p*, in Inhibition of Pancreatic Carcinoma Cell Proliferation

**DOI:** 10.3390/ijms15046314

**Published:** 2014-04-14

**Authors:** Chunlong Li, Xuefei Du, Sheng Tai, Xiangyu Zhong, Zhidong Wang, Zhanliang Hu, Lei Zhang, Pengcheng Kang, Daolin Ji, Xingming Jiang, Qingxin Zhou, Ming Wan, Guixing Jiang, Yunfu Cui

**Affiliations:** 1 Department of Hepatopancreatobiliary Surgery, Second Affiliated Hospital of Harbin Medical University, Harbin 150081, China; E-Mails: chunlong81@163.com (C.L.); shengtai75@163.com (S.T.); xiangyuzhong76@126.com (X.Z.); zhidongwang74@126.com (Z.W.); zhanlianghu65@163.com (Z.H.); leizhang74@163.com (L.Z.); pengchengkang84@163.com (P.K.); daolinji84@163.com (D.J.); xingmingjiang87@163.com (X.J.); qingxinzhou88@yeah.net (Q.Z.); mingwan84@163.com (M.W.); 2 Department of Laboratory Diagnostics, Fourth Affiliated Hospital of Harbin Medical University, Harbin 150001, China; E-Mail: duxuefei82@163.com; 3 Department of Hepatopancreatobiliary Surgery, Second Affiliated Hospital of Zhejiang University School of Medicine, Hangzhou 310009, China; E-Mail: guixingjiang84@163.com

**Keywords:** *miR-96-5p*, *miR-182-5p*, *GPC1*, PC

## Abstract

To determine the relationships between *miR-96-5p*/*-182-5p* and *GPC1* in pancreatic cancer (PC), we conducted the population and *in vitro* studies. We followed 38 pancreatic cancer patients, measured and compared the expression of *miR-96-5p*/*-182-5p*, *GPC1*, characteristics and patients’ survival time of different *miR-96-5p*/*-182-5p* expression levels in PC tissues. In an *in vitro* study, we investigated the proliferation, cycle and apotosis in cells transfected with mimics/inhibitors of the two miRNAs, and determine their effects on *GPC1* by dual-luciferase assay. In the follow-up study, we found that the expressions of *miR-96-5p*/*-182-5p* were lower/higher in PC tissues; patients with lower/higher levels of *miR-96-5p*/*-182-5p* suffered poorer characteristics and decreased survival time. In the *in vitro* study, the expressions of *miR-96-5p*/*-182-5p* were different in cells. Proliferation of cells transfected with *miR-96-5p* mimics/inhibitors was lower/higher in Panc-1/BxPC-3; when transfected with *miR-182-5p* mimics/inhibitors, proliferation of cells were higher/lower in AsPC-1/Panc-1. In a cell cycle study, panc-1 cells transfected with *miR-96-5p* mimics was arrested at G0/G1; BxPC-3 cells transfected with *miR-96-5p* inhibitors showed a significantly decrease at G0/G1; AsPC-1 cells transfected with *miR-182-5p* mimics was arrested at S; Panc-1 cells transfected with *miR-182-5p* inhibitors showed a decrease at S. *MiR-96-5p* mimics increased the apoptosis rate in Panc-1 cells, and its inhibitors decreased the apoptosis rate in BxPC-3. Dual luciferase assay revealed that *GPC1* was regulated by *miR-96-5p*, not *-182-5p*. We found that *miR-96-5p*/*-182-5p* as good markers for PC; *miR-96-5p*, rather than *-182-5p*, inhibits *GPC1* to suppress proliferation of PC cells.

## 1. Introduction

Pancreatic cancer (PC) usually refers to the pancreatic ductal adenocarcinoma, with high incidences of tumor recurrence and metastasis. PC is currently the fourth most common cause of cancer-related mortality in Western societies, and an overall five-year survival rate among patients with PC is less than 5% [1,2].

Heparan sulfate proteoglycan glypican-1 (*GPC1*) and *GPC3* are members of heparan sulfate proteoglycans (HSPG) family, which are ubiquitous proteins attached to the extracytoplasmic surface of the cell membrane. Recently, *GPC1* and *GPC3* were found to be predictor and target for some cancers [3,4], including PC [5–8]. It has been verified that miRNAs, *miR-96-5p* and *-182-5p*, could regulate expression of *GPC3* [9,10]. However, the regulators of *GPC1* are still unknown.

Recently, a new batch of endogenous small non-coding regulatory RNAs, the microRNAs (miRNAs), has been paid attention to the cancer development. In human carcinoma tissue, emerging evidence has shown that miRNA-induced regulation contributes to maintain a biological process of the proliferation, differentiation, and apoptosis [11]. MiRNAs are key negative regulators of gene expression by binding to the 3′-untranslated region (3′-UTR) of corresponding target messenger RNAs (mRNAs) [12]. Recently, *miR-96-5p* and *-182-5p*, were found as the upstream regulators for some cancers, such as prostate cancer [13], breast cancer [14], bladder cancer [15], and medulloblastoma [16]. Specifically, *miR-96-5p* was found down regulated in PC [17,18], but *miR-182-5p* has not been studied in PC yet. In addition, it is still unknown with whether these miRNAs could also regulate the expression of *GPC1*.

Based on the close relationships between *GPC3*, *miR-96-5p*, *-182-5p*, and cancers, we made a hypothesis that *miR-96-5p* and/or *-182-5p* might regulate *GPC1* expression in the development of PC.

Therefore, in this study we aim to clarify three issues: (1) whether *miR-96-5p* and/or *-182-5p* could regulate *GPC1* in PC; (2) whether *miR-182-5p* plays a role in PC; and (3) to validate the effect of *miR-96-5p* in PC.

## 2. Results and Discussion

### 2.1. The Correlations between Expression of miR-96-5p, -182-5p and GPC1 Expression, Clinicopathological Characteristics of PC

**Figure 1.**
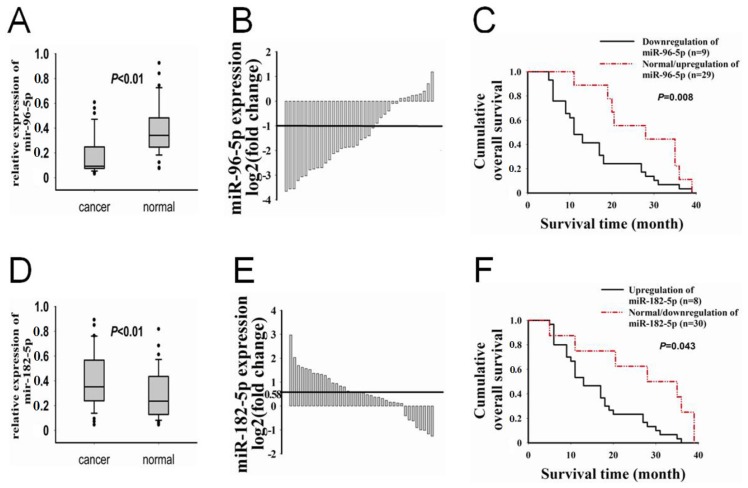
Analysis of *miR-96-5p* and *miR-182-5p* expression in human PC tissues and survival time of PC patients. (**A**) Real-time PCR analysis of *miR-96-5p* expression in human pancreatic cancer tissues (*n* = 38) and matched adjacent noncancerous pancreatic tissues (*n* = 38). PC, pancreatic cancer tissues; NP, adjacent noncancerous pancreatic tissues; (**B**) *miR-96-5p* expression was examined in 38 pairs of PC tissues and NP tissues by qRT-PCR. Data were presented as log2 of fold change of PC relative to NP. The cases below the line (log2 = −1) showed a >50% decrease in the *miR-96-5p* expression level; (**C**) Kaplan-Meier curves of the overall survivals of 38 pancreatic cancer patients were scored as *miR-96-5p* downregulation group (*n* = 29) and normal/upregulation group (*n* = 9); (**D**) Real-time PCR analysis of *miR-182-5p* expression in human pancreatic cancer tissues (*n* = 38) and matched adjacent noncancerous pancreatic tissues (*n* = 38); (**E**) *miR-182-5p* expression was examined in 38 pairs of PC tissues and NP tissues by qRT-PCR. Data were presented as log2 of fold change of PC relative to NP. The cases above the line (log2 = 0.58) showed a >150% increase in the *miR-182-5p* expression level; (**F**) Kaplan-Meier curves of the overall survivals of 38 pancreatic cancer patients were scored as *miR-182-5p* upregulation group (*n* = 30) and normal/downregulation group (*n* = 8). The *p*-values are shown with the use of log-rank test. All data are presented as mean ± SD.

In this study, the expression levels of *miR-96-5p* in the 38 (100%) pairs of PC tissue and matched non-tumor adjacent tissue were determined by qRT-PCR, with snRNA U6 as the endogenous control. We found that *miR-96-5p* expression was significantly down regulated in PC samples (*p* < 0.01). The median *miR-96-5p* expression level in PC tissues was two-fold lower than that of the non-tumor adjacent tissues (median expression 0.176 *vs*. 0.387, respectively) (Figure 1A). Among the 38 PC patients, 23 (60.53%) showed a reduction in the *miR-96-5p* level (a reducted *miR-96-5p* level was defined as expression decreased more than 50% relative to the matched non-tumor adjacent tissues, which was >1 cm from the pancreatic carcinoma) (Figure 1B). We then analyzed the correlations between *miR-96-5p* expression and clinicpathological characteristics. The patients with lower levels of *miR-96-5p* expression tended to suffer larger tumor sizes (larger tumor size was defined as ≥2 cm; *p* = 0.029; Table 1) and poorer differentiation (*p* = 0.034; Table 1), and there was no significant correlation between *miR-96-5p* expression and other clinicpathological characteristics. Further, to determine the relationship between *miR-96-5p* expression and prognosis of PC, we analyzed the data of the three-year-survival time in PC patients. The Kaplan-Meier analysis showed that down regulated expression of *miR-96-5p* was associated with a decreased survival (Figure 1C). Specifically, the median overall survival time was 16.0 and 27.0 months in *miR-96-5p* downregulation and normal/upregulation groups, respectively (*p* = 0.008, log-rank test).

In contrast with *miR-96-5p*, expression of *miR-182-5p* was up regulated in tissues of PC patients (*p* < 0.01). The median *miR-182-5p* expression level in PC tissues and non-tumor adjacent tissues was 0.403 *vs*. 0.294, respectively (Figure 1D). Among the 38 PC patients, 17 (44.73%) showed a rise in the *miR-182-5p* level (a reducted *miR-96-5p* level was defined as expression decreased more than 50% relative to the matched non-tumor adjacent tissues) (Figure 1E). The patients with higher levels of *miR-182-5p* expression tended to suffer poorer differentiation (*p* = 0.007; Table 1). The Kaplan-Meier analysis showed that up regulated expression of *miR-182-5p* was associated with a decreased survival (Figure 1F). The median overall survival time was 16.0 and 26.0 months in *miR-182-5p* upregulation and normal/downregulation groups, respectively (*p* = 0.043, log-rank test).

To confirm the correlations between the expression of *miR-96-5p* and *-182-5p* and *GPC1* expression in PC tissue samples, we measured *GPC1* protein levels in 38 PC tissues using immunohistochemistry. The expressions of *GPC1* were defined as normal/underexpression (−/+) and overexpression (++/+++). *GPC1* of 25 PC tissues were overexpressed in 38 cases (65.79%), and we observed an inverse correlation between the expression of *miR-96-5p* and *GPC1* protein in PC tissue samples (*r* = −0.381, *p* = 0.018) (Table 2). However, the correlation between the expression of *miR-182-5p* and *GPC1* protein was not significant.

### 2.2. The Expression of miR-96-5p and -182-5p in Cell Lines and Their Effects on PC Cell Proliferation

**Figure 2.**
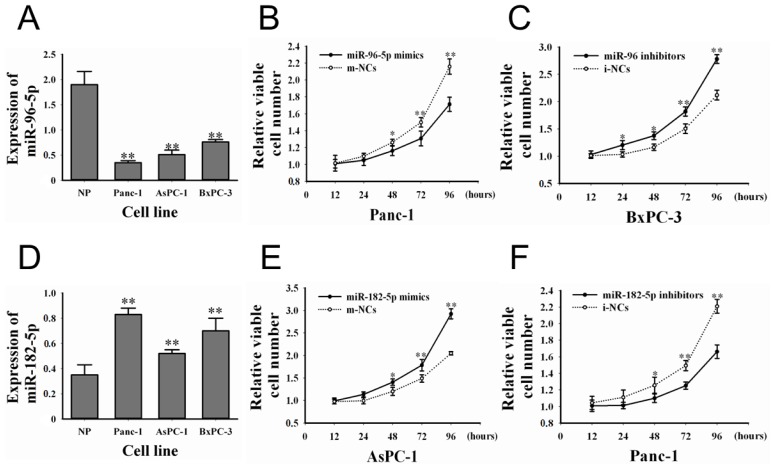
The expression of *miR-96-5p* and *-182-5p* in cell lines and their effects on PC cell proliferation. (**A**) The expression level of *miR-96-5p* in three pancreatic cancer cell lines (Panc-1, AsPC-1, and BxPC-3) and normal pancreatic tissues; (**B**) Upregulation of *miR-96-5p* inhibited proliferation in Panc-1; (**C**) Downregulation of *miR-96-5p* promoted proliferation in BxPC-3; (**D**) The expression level of *miR-182-5p* in three pancreatic cancer cell lines (Panc-1, AsPC-1, and BxPC-3) and normal pancreatic tissues; (**E**) Upregulation of *miR-182-5p* promoted proliferation in AsPC-1; (**F**) Downregulation of *miR-182-5p* inhibited proliferation in Panc-1. All data from three separate experiments are presented as mean ± SD. * *p* < 0.05; ** *p* < 0.01.

The expression levels of *miR-96-5p* in the PC cell lines were determined by qRT-PCR (snRNA U6 as the endogenous control). As there was no normal pancreatic cell line, we randomly chose three samples of normal pancreatic tissue as the control. We also found that, compared to normal pancreatic tissues, the expression of *miR-96-5p* was lower in all three PC cell lines (Panc-1, 0.35 ± 0.04-fold; AsPC-1, 0.51 ± 0.09-fold; BxPC-3, 0.76 ± 0.05-fold, Figure 2A). In contrast with *miR-96-5p*, expression of *miR-182-5p* was higher in all three cell lines (Panc-1, 0.83 ± 0.05-fold; AsPC-1, 0.52 ± 0.03-fold; BxPC-3, 0.70 ± 0.10-fold, Figure 2D). Based on the differences of both miRNAs expression in three cell lines, we selected the Panc-1, BxPC-3 for *miR-96-5p* and Panc-1, AsPC-1 for *miR-182-5p* in PC cell experiments.

The changed expressions of *miR-96-5p* and *-182-5p* in PC suggested to investigate whether the both miRNAs have effects on the PC proliferation. In proliferation assay, we found that proliferation of cells transfected with *miR-96-5p* mimics was lower than that of cells transfected with m-NCs in Panc-1 (Figure 2B), and reducing *miR-96-5p* expression by *miR-96-5p* inhibitors promoted cell proliferation in BxPC-3 (Figure 2C). In *miR-182-5p* study, we found that restoration of *miR-182-5p* expression by *miR-182-5p* mimics promoted cell proliferation in AsPC-1 (Figure 2E), and reduced *miR-182-5p* expression by *miR-182-5p* inhibitors could inhibit cell proliferation in Panc-1 cells (Figure 2F).

### 2.3. Effects of miR-96-5p and -182-5p on Cell Cycle and Apoptosis in PC Cells

**Figure 3.**
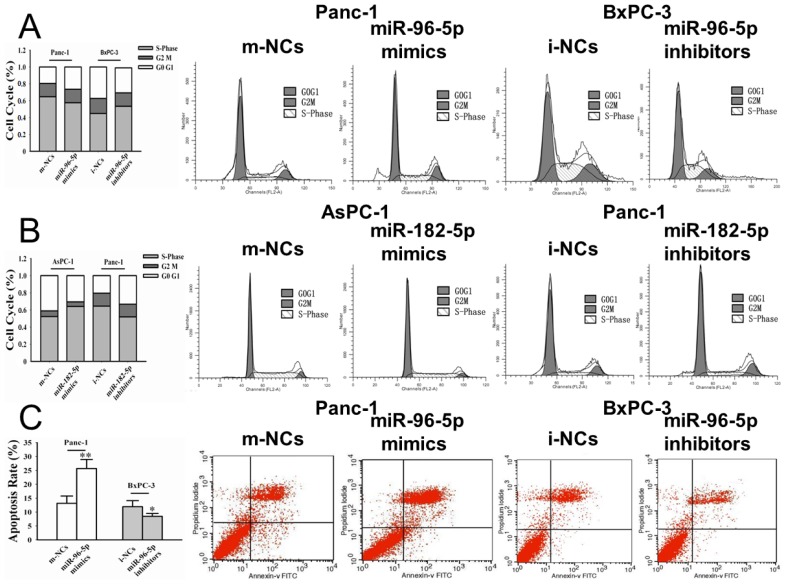
Effects of *miR-96-5p* and *-182-5p* on cell cycle and apoptosis in PC cells. (**A**) Flow cytometric analysis of indicated PC cancer cells transfected with *miR-96-5p* mimics, inhibitors or their respective negative controls (NCs) in Panc-1 and BxPC-3. *MiR-96-5p* induced cell cycle arrest at G1/G0 phase; (**B**) Flow cytometric analysis of indicated PC cancer cells transfected with *miR-182-5p* mimics, inhibitors or their respective NCs in AsPC-1 and Panc-1. *MiR-182-5p* induced cell cycle arrest at S phase; (**C**) PC cancer cells transfected with *miR-96-5p* mimics, inhibitors or their respective NCs in Panc-1 and BxPC-3, and the apoptosis was measured by flow cytometry. * *p* < 0.05; ** *p* < 0.01.

Knowing that *miR-96-5p* obviously inhibited PC cell proliferation *in vitro*, we determined whether *miR-96-5p* could influence cell cycle progression in PC cells. The cell cycle distribution of Panc-1 cells transfected with *miR-96-5p* mimics showed a significant increase in the proportion of cells in the G1/G0 phase and a decrease in the proportion of cells in the S phase as compared to that of the m-NCs (mimics-negative controls) (*p* < 0.01) (Figure 3A). In contrast, the cell cycle distribution analysis of BxPC-3 cells after transfection of *miR-96-5p* inhibitors showed a significantly decrease proportion of cells in the G0/G1 phase and increased proportion of cells in the S phase, compared to i-NCs-transfected cells (inhibitors-negative controls) (*p* < 0.01) (Figure 3A). For *miR-182-5p*, the cell cycle distribution of AsPC-1 cells showed that the proportion of G0/G1 phase dropped more significantly in cells transfected with *miR-182-5p* mimics than in m-NCs, and the proportion of S phase rised sharply (*p* < 0.01) (Figure 3B). After transfected with *miR-182-5p* inhibitors, the proportion of G0/G1 phase went up and the proportion of S phase went down in Panc-1 (*p* < 0.01) (Figure 3B).

For the effects of *miR-96-5p* on apoptosis, we found that *miR-96-5p* mimics significantly increased the apoptosis rate in Panc-1 as compared to that of the m-NCs (25.68% ± 3.25% *vs*. 13.16% ± 2.61%), and *miR-96-5p* inhibitors decreased cell apoptosis in BxPC-3 as compared to that of the i-NCs (8.46% ± 1.04% *vs*. 11.92% ± 2.18%) (Figure 3C). However, *miR-182-5p* had no significant effect on apoptosis in panc-1 and AsPC-1 cells at 48 h after transfection.

### 2.4. GPC1 Might Be a Direct Downstream Target for miR-96-5p

**Figure 4.**
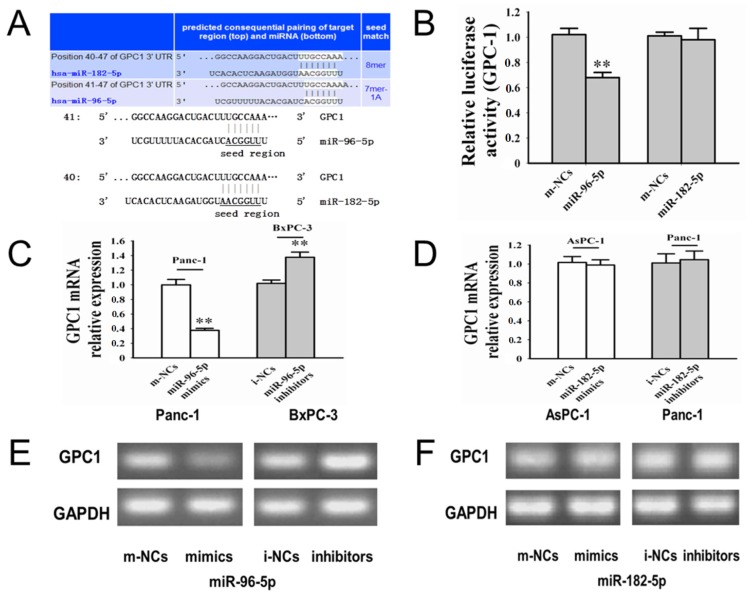
Effects of *miR-96-5p* and *-182-5p* on *GPC1* expression. (**A**) The *miR-96-5p* and the *miR-96-5p*-binding site in the 3′-UTR of *GPC1*. The *miR-182-5p* and the *miR-182-5p*-binding site in the 3′-UTR of *GPC1*; (**B**) Luciferase reporter assay with cotransfection of *GPC1* 3′-UTR and *miR-96-5p*, *miR-182-5p*, and their respective NCs in HEK-293T cells; (**C**) The expression level of *GPC1* mRNA was down-regulated by *miR-96-5p*; (**D**) The expression level of *GPC1* mRNA was not significantly regulated by *miR-182-5p* using qRT-PCR; (**E**) Western blot results of *GPC1* protein in Panc-1 and BxPC-3 transfected with *miR-96-5p* mimics, *miR-96-5p* inhibitors and their respective NCs at 48 h; (**F**) The expression level of *GPC1* protein was not significantly regulated by *miR-182-5p*. All data from three separate experiments are presented as mean ± SD. ** *p* < 0.01.

As shown in Figure 4B, the relative luciferase activity in cells cotransfected with *miR-96-5p* mimics was significantly decreased after 48 h (*p* < 0.01). The reduction in the luciferase activity with *miR-182-5p* mimics was not significant relative to m-NCs (*p* = 0.713). Above results suggested that *miR-96-5p*, not *miR-182-5p*, could directly bind to the 3′-UTR of human *GPC1*. We examined the *GPC1* mRNA levels in three cells post-transfection by qRT-PCR. After 48 h of transfection, we found that mRNA level of *GPC1* to have been suppressed by *miR-96-5p* mimics in Panc-1 (*p* < 0.01) (Figure 4C), and mRNA level of *GPC1* increased in BxPC-3 cells transfected *miR-96-5p* inhibitors (*p* < 0.01) (Figure 4C). We also found that *miR-96-5p* mimics reduced *GPC1* protein levels in panc-1, and *GPC1* protein levels increased in BxPC-3 cells transfected *miR-96-5p* inhibitors at 48 h (Figure 4E). After transfection of *miR-182-5p* mimics or inhibitors, significant changes were not observed in *GPC1* mRNA levels and *GPC1* protein levels (Figure 4D,F). These data suggest that *miR-96-5p* negatively regulates *GPC1* protein expression through mRNA degradation and translational repression, and *GPC1* could not be the target of *miR-182-5p*.

### 2.5. Discussion

#### 2.5.1. Whether *miR-96-5p* and/or *-182-5p* Could Regulate *GPC1* in PC

Previous studies have indicated that *GPC1* is commonly overexpressed in human PC, and it directly contributes to the carcinogenesis and development of PC by controlling cell proliferation [8]. As previous study indicated that *miR-96-5p* and *-182-5p* could regulate *GPC3* expression, which is also the member of HBGFs, the same as *GPC1* [8–10], we took advantage of bioinformatics to predict the putative target genes of *miR-96-5p* and *-182-5p* by using TargetScan, miRanda, and PicTar algorithms, which also suggest that *GPC1* might be a potential target for *miR-96-5p* and *-182-5p* (Figure 4A). Then, we did a series of tests to determine the relationships between *miR-96-5p*, *-182-5p* and *GPC1* in three PC cell lines. We measured the *miR-96-5p* and *miR-182-5p* expression in Panc-1, AsPC-1, BxPC-3, and found *miR-96-5p* was lower in Panc-1 and higher in BxPC-3 when compared with normal pancreatic tissues; and *miR-182-5p* was lower in AsPC-1 and higher in Panc-1. To verify whether *GPC1* is a direct target of *miR-96-5p*, we performed dual-luciferase assay, Quantitative real-time PCR (qRT-PCR), and Western blot analysis in Panc-1 and BxPC-3 cell lines, respectively; and confirmed that *miR-96-5p* could directly regulate *GPC1* expression; but *miR-182-5p* failed. In addition, the transfection study showed that significant negative changed expression of *GPC1* with the *miR-96-5p* treatment in a time dependent manner; but not found in *miR-182-5p* treatment group.

In this study, we firstly found that *GPC1* was regulated by *miR-96-5p* rather than *miR-182-5p*. Indeed, *miR-96-5p* has other targets in PC, such as KRAS [17,18]. Our study implies that may be more molecular mechanisms exits in the PC development. Moreover, as miRNAs is the upstream of many target proteins in cancer development, anti cancer drugs targeting in miRNA might have better therapeutic effects in specific cancer.

#### 2.5.2. Whether *miR-182-5p* Could Play a Role in PC

In previous study, *miR-182-5p* was reported to be over-expressed in some cancers, such as prostate cancer [13], bladder cancer [15], however, *miR-182-5p* has not been studied in PC yet. Our study suggested that *miR-182-5p* is a good marker for development and survival time of PC in the population study. Expression of *miR-182-5p* was up regulated in tissues of PC patients. Patients with higher levels of *miR-182-5p* expression tended to suffer poorer differentiation. Up-regulated expression of *miR-182-5p* was associated with a decreased survival in our PC patients. Additionally, in *in vitro* study, the increased *miR-182-5p* expression could increase the proliferation of PC cell lines. PC cells in high expression of *miR-182-5p* were cell cycle arrest at S phase, suggesting that up regulation of *miR-182-5p* in PC may cause the cancer cells to divide and grow more quickly. However, we did not find the correlation between expression of *miR-182-5p* and *GPC1* protein, nor detect the effects of *miR-182-5p* treatment on *GPC1* protein expression. It should have some other molecular targets, which need further investigation. In this study, we firstly found that *miR-182-5p* could be a biomarker in PC.

#### 2.5.3. The Validation of the *miR-96-5p* Effects on PC

Previous studies found that *miR-96-5p* inhibit cell proliferation in prostate cancer and hepatoma cells [19,20] and only one report in PC [18]. Our findings also showed that *miR-96-5p* could significantly suppress PC cell proliferation *in vitro* and cell cycle analysis and apoptosis assay indicated that *miR-96-5p* can induce Panc-1 cells cycle arrest at the G1/G0 phase and increase the apoptosis rate of Panc-1 cells, suggesting that down regulation of *miR-96-5p* in PC may cause the cancer cells to divide and grow more quickly.

We also measured the *miR-96-5p* expression in the PC tissue of PC patients. In the follow-up study, the down regulated *miR-96-5p* expressions in the pancreatic samples were consistent with previous studies [17,18]. In addition, lower *miR-96-5p* expressions were related with the poorer clinicopathological characteristics of PC and survival time in PC patients. These findings indicated a validated relationship between *miR-96-5p* and pancreatic cancer. To explore whether *miR-96-5p* or *-182-5p* influences the *GPC1* expression, we further analyzed the correlations between expression of *miR-96-5p* and *GPC1* protein in 38 PC tissue samples and found only expression of *miR-96-5p* was correlated with *GPC1* protein. These findings also suggested a close relationship between *miR-96-5p* and *GPC1* in PC.

## 3. Experimental Section

### 3.1. Patients and Tissue Samples

A total of 38 pairs of the pancreatic ductal adenocarcinoma and corresponding non-tumor adjacent tissue samples from PC patients were obtained at the Second Affiliated Hospital of Harbin Medical University from March 2008 to August 2011. The clinical characteristics of the patients were collected, including tumor location, histological type, differentiation grade, lymph node invasion, and TNM staging. We followed the all PC patients included for three-year survival. The study was approved by the Research Ethics Committee of Harbin Medical University.

### 3.2. Immunohistochemistry Staining and Staining Evaluation

*GPC1* protein expression was detected immunohistochemically using paraffin-embedded tissues specimens from 38 PC patients according to the methods in our previous studies [21].

### 3.3. Cell Lines and Transfection

Human PC cell lines Panc-1, AsPC-1 and BxPC-3 and human embryonic kidney 293 T cell line (HEK293T), which were obtained from the Chinese Academy of Sciences Shanghai Branch Cell Bank (Shanghai, China), were propagated in DMEM (Beyotime, Shanghai, China) supplemented with 10% fetal bovine serum. The cells were maintained at 37 °C in an atmosphere of humidified air with 5% CO_2_. Transfection was performed with Lipofectamine 2000 Reagent (Invitrogen, Carlsbad, CA, USA) according to the manufacturer instructions. *MiR-96-5p*, *-182-5p* mimics, mimic negative controls (m-NCs), *miR-96-5p*, *-182-5p* inhibitors, and inhibitor negative controls (i-NCs) were purchased from Ribobio (Guangzhou, China). A final concentration of 50 nM of mimics or 100 nM of inhibitors and their respective negative controls (NCs) were used for each transfection in proliferation, cell cycle and apoptosis experiments.

### 3.4. RNA Extraction and qRT-PCR

Total RNA samples were isolated from primary tissues or from cell lines using TRIzol (Invitrogen, Carlsbad, CA, USA) and reverse transcribed to cDNA using PrimeScript Regent Kit (Takara, Tokyo, Japan). Quantitative real-time PCR was performed using SYBR green kit (Takara, Tokyo, Japan) and the ABI 7500 Real-time PCR system (Applied Biosystems, Foster City, CA, USA) according to the manufacturer’s instructions. Primers were obtained from RiboBio (RiboBio, Guangzhou, China) and listed in Supplementary Table S1. All assays were performed at least in triplicate. The relative expression was normalized to that of internal control snRNA U6 or GAPDH and calculated using 2^−ΔΔ^*^C^*^t^ method [22]. Changes in the expression of PC were found to be relative to the non-tumorous controls (non-tumorous tissue adjacent to tumors).

### 3.5. Cell Proliferation Assay

The cell proliferation was indirectly assayed using the CCK-8 kit (Beyotime, Shanghai, China), which stains living cell according to the manufacturer’s instructions. Five thousand cells were seeded in 96-well plates and transfected with mimics, inhibitors, or their respective NCs the next day. CCK-8 solution was used to measure cell viability at 48 h after transfection. The absorbance of each well was measured with a microplate reader set at 450 nm. All experiments were performed in triplicate.

### 3.6. Cell Cycle Assay and Apoptosis Analysis

For the cell cycle analysis, cells were harvested by trypsinization, washed twice using cold PBS and fixed in 70% ethanol overnight at −20 °C. Then cells were treated with DNA staining solution containing 3.4 mM Tris-Cl (pH 7.4), propidium iodide, 0.1% triton X-100 buffer and 100 μg/mL RNase A. Cell cycle analysis was performed with FACS flow cytometry. For apoptosis analysis, 48 h after transfection, the cells were collected, washed twice with cold phosphate buffered saline (PBS) and re-suspended in binding buffer at a cell density of 1 × 10^6^/mL. Cells were then stained with Annexin V-FITC (BioVision, San Francisco, CA, USA) and propodium iodide according to the manufacturer’s instructions (Beyotime, Shanghai, China). The signal was acquired by a FACS Calibur flow cytometer (BD Biosciences, Mississauga, ON, Canada) and was analyzed with Cellquest software (BD Biosciences, Mississauga, ON, Canada). Each sample was run in triplicate.

### 3.7. Dual-Luciferase Reporter Assay

Target sequences of *GPC1* 3′-UTR (Figure 4A) were cloned into the dual-luciferase reporter vectors CHECK-*GPC1*-96 and CHECK-*GPC1*-182, which was constructed by RiboBio (RiboBio Co., Guangzhou, China). HEK-293T cells were plated into 24-well plates with 50%–60% confluence 24 h before transfection. A mixture of 50 nM *miR-96-5p* or *-182-5p* mimics, and 0.5 μg CHECK-*GPC1*-96 or CHECK-*GPC1*-182 report vector was transfected into cells using Lipofectamine 2000 reagent (Invitrogen, Carlsbad, CA, USA). After 48 h, luciferase activity levels were measured using a dual-luciferase reporter assay system (Promega, Madison, WI, USA) and normalized by dividing firefly luciferase activity by that of Renilla luciferase according to the manufacturers’ instructions. Each transfection was performed in triplicate.

### 3.8. Western Blot

Total proteins samples were extracted from primary tissues or from cell lines using lysis buffer containing phenylmethyl sulfonyfluoride (PMSF). The samples were mixed with loading buffer and denatured, separated by electrophoresis in a 10% SDS-PAGE gel, and then transferred to polyvinylidene fluoride (PVDF) membranes. The membranes were blocked with 5% nonfat milk for 2 h, incubated with anti-*GPC1* antibody (Abcam, Cambridge, UK) or anti-GAPDH antibody (Abcam, Cambridge, UK) overnight at 4 °C. Signals were revealed after incubation with anti-rabbit IgG secondary antibody coupled to peroxidase by using ECL.

### 3.9. Statistical Analysis

The data were presented as means ± standard deviation (SD). The *miR-96-5p* and *-182-5p* expressions were compared in pancreatic cancer tissues and cells by the unpaired Student’s *t*-test. The relationships between *miR-96-5p*, *-182-5p*, and clinicopathologic parameters were evaluated by χ^2^ test. The survival rates for *miR-96-5p* and *-182-5p* expressions were estimated by using the Kaplan-Meier method and the difference in survival curves were tested by log-rank test. The relationships between *miR-96-5p*, *-182-5p*, and *GPC1* expression were explored by Spearman’s correlation. All statistical analyses were carried out by SPSS13.0 software (SPSS, Chicago, IL, USA). *p* < 0.05 was considered statistically significant.

## 4. Conclusions

Our study suggested that both *miR-96-5p* and *-182-5p* could be the up stream of PC development. The structure of *miR-96-5p* and *-182-5p* are similar, however, their function differs significantly as the data in our study showed above. To study the exact effects of the each miRNA, further investigations on the differences of functions, down stream targets and bio-effects between them are needed.

In conclusion, our study found that both *miR-96-5p* and *-182-5p* are both good markers for PC; *miR-96-5p*, rather than *-182-5p*, could directly inhibit *GPC1* to suppress proliferation of PC cells.

## Supplementary Information



## Figures and Tables

**Table 1.** Correlations between *miR-96-5p*, *miR-182-5p*, and various clinicopathological characteristics of pancreatic cancer (PC) patients.

Clinicopathological Characteristics		*miR-96-5p*		*miR-182-5p*	

		Down (%)	Normal/Up (%)	Up (%)	Normal/Down (%)
Gender	Male	21 (75.0)	7 (25.0)	22 (78.6)	6 (21.4)
	Female	8 (80.0)	2 (20.0)	8 (80.0)	2 (20.0)
Age (years)	<60	16 (72.7)	6 (27.3)	17 (77.3)	5 (22.7)
	≥60	13 (81.2)	3 (18.8)	13 (81.3)	3 (18.7)
Serum CA19-9	Negative	5 (71.4)	2 (28.6)	5 (71.4)	2 (28.6)
	Positive	24 (77.4)	7 (22.6)	25 (80.6)	6 (19.4)
Tumor size	<2	18 (66.7)	9 (33.3) *	21 (77.8)	6 (22.2)
	≥2	11 (100.0)	0 (0.0)	9 (81.8)	2 (18.2)
Histological differentiation	Well	14 (63.6)	8 (36.4) *	14 (63.6)	8 (36.4) *
	Poorly	15 (93.7)	1 (6.3)	16 (100.0)	0 (0.0)
Pancreatitis	No	21 (77.8)	6 (22.2)	23 (85.2)	4 (14.8)
	Yes	8 (72.7)	3 (27.3)	7 (63.6)	4 (36.4)
Regional lymph node metastasis	No	23 (74.2)	8 (25.8)	24 (77.4)	7 (22.6)
	Yes	6 (85.7)	1 (14.3)	6 (85.7)	1 (14.3)
TNM staging	I-IIA	23 (74.2)	8 (25.8)	24 (77.4)	7 (22.6)
	IIB	6 (85.7)	1 (14.3)	6 (85.7)	1 (14.3)

* *p* < 0.05.

**Table 2.** Correlations between *miR-96-5p*, *miR-182-5p*, and *GPC1* protein of PC patients.

*GPC1*	*n*	*miR-96-5p*		Correlation		*miR-182-5p*		Correlation	

		down	up/Normal	*r*	*p*	up	Normal/down	*r*	*p*
Normal/underexpression(−/+)	13	7	6	−0.381	0.018	11	2	0.100	0.549
Overexpression(++/+++)	25	22	3			19	6		

*p* for Spearman’s correlation.

## References

[1] Vincent A., Herman J., Schulick R., Hruban R.H., Goggins M. (2011). Pancreatic cancer. Lancet.

[2] Hidalgo M. (2010). Pancreatic cancer. N. Engl. J. Med.

[3] Feng M., Gao W., Wang R., Chen W., Man Y.G., Figg W.D., Wang X.W., Dimitrov D.S., Ho M. (2013). Therapeutically targeting glypican-3 via a conformation-specific single-domain antibody in hepatocellular carcinoma. Proc. Natl. Acad. Sci. USA.

[4] Aikawa Aikawa T., Whipple C.A., Lopez M.E., Gunn J., Young A., Lander A.D., Korc M. (2008). Glypican-1 modulates the angiogenic and metastatic potential of human and mouse cancer cells. J. Clin. Investig.

[5] Duan L., Hu X.Q., Feng D.Y., Lei S.Y., Hu G.H. (2013). GPC-1 may serve as a predictor of perineural invasion and a prognosticator of survival in pancreatic cancer. Asian J. Surg./Asian Surg. Assoc.

[6] Mounajjed T., Zhang L., Wu T.T. (2013). Glypican-3 expression in gastrointestinal and pancreatic epithelial neoplasms. Hum. Pathol.

[7] Lee S., Reha J.L., Tzeng C.W., Massarweh N.N., Chang G.J., Hetz S.P., Fleming J.B., Lee J.E., Katz M.H. (2013). Race does not impact pancreatic cancer treatment and survival in an equal access federal health care system. Ann. Surg. Oncol.

[8] Whipple C.A., Young A.L., Korc M. A. (2012). KrasG12D-driven genetic mouse model of pancreatic cancer requires glypican-1 for efficient proliferation and angiogenesis. Oncogene.

[9] Jalvy-Delvaille S., Maurel M., Majo V., Pierre N., Chabas S., Combe C., Rosenbaum J., Sagliocco F., Grosset C.F. (2012). Molecular basis of differential target regulation by miR-96 and miR-182: The Glypican-3 as a model. Nucleic Acids Res.

[10] Moskwa P., Buffa F.M., Pan Y., Panchakshari R., Gottipati P., Muschel R.J., Beech J., Kulshrestha R., Abdelmohsen K., Weinstock D.M. (2011). miR-182-mediated downregulation of BRCA1 impacts DNA repair and sensitivity to PARP inhibitors. Mol. Cell.

[11] Hwang H.W., Mendell J.T. (2006). MicroRNAs in cell proliferation, cell death, and tumorigenesis. Br. J. Cancer.

[12] Lee R.C., Ambros V. (2001). An extensive class of small RNAs in Caenorhabditis elegans. Science.

[13] Tsuchiyama K., Ito H., Taga M., Naganuma S., Oshinoya Y., Nagano K., Yokoyama O., Itoh H. (2013). Expression of microRNAs associated with Gleason grading system in prostate cancer: *miR-182-5p* is a useful marker for high grade prostate cancer. Prostate.

[14] Guttilla I.K., White B.A. (2009). Coordinate regulation of FOXO1 by miR-27a, miR-96, and miR-182 in breast cancer cells. J. Biol. Chem.

[15] Guo Y., Liu H., Zhang H., Shang C., Song Y. (2012). miR-96 regulates FOXO1-mediated cell apoptosis in bladder cancer. Oncol. Lett.

[16] Weeraratne S.D., Amani V., Teider N., Pierre-Francois J., Winter D., Kye M.J., Sengupta S., Archer T., Remke M., Bai A.H. (2012). Pleiotropic effects of miR-183~96~182 converge to regulate cell survival, proliferation and migration in medulloblastoma. Acta Neuropathol.

[17] Tanaka M., Suzuki H.I., Shibahara J., Kunita A., Isagawa T., Yoshimi A., Kurokawa M., Miyazono K., Aburatani H., Ishikawa S. (2013). EVI1 oncogene promotes KRAS pathway through suppression of microRNA-96 in pancreatic carcinogenesis. Oncogene.

[18] Yu S., Lu Z., Liu C., Meng Y., Ma Y., Zhao W., Liu J., Yu J., Chen J. (2010). miRNA-96 suppresses KRAS and functions as a tumor suppressor gene in pancreatic cancer. Cancer Res.

[19] Haflidadottir B.S., Larne O., Martin M., Persson M., Edsjo A., Bjartell A., Ceder Y. (2013). Upregulation of miR-96 Enhances Cellular Proliferation of Prostate Cancer Cells through FOXO1. PLoS One.

[20] Xu D., He X., Chang Y., Xu C., Jiang X., Sun S., Lin J. (2013). Inhibition of miR-96 expression reduces cell proliferation and clonogenicity of HepG2 hepatoma cells. Oncol. Rep.

[21] Zhong X.Y., Yu J.H., Zhang W.G., Wang Z.D., Dong Q., Tai S., Cui Y.F., Li H. (2012). MicroRNA-421 functions as an oncogenic miRNA in biliary tract cancer through down-regulating farnesoid X receptor expression. Gene.

[22] Livak K.J., Schmittgen T.D. (2001). Analysis of relative gene expression data using real-time quantitative PCR and the 2^−ΔΔ^*^C^*^t^ Method. Methods.

