# Supplementary material for: GPC1 Regulated by miR-96-5p, Rather than miR-182-5p, in Inhibition of Pancreatic Carcinoma Cell Proliferation
Source: Int J Mol Sci. 2014 Apr 14;15(4):6314–27. doi: 10.3390/ijms15046314 (PMC4013630; doi:10.3390/ijms15046314)
Supplement: Supplementary file 1 [file ijms-15-06314-s001.pdf]

Supplementary Information

Table S1. Primer sequences.

Name	Primer sequences	
	Forward	Reverse
miR-96-5p	5'-TTTGGCACTAGCACAT-3'	5'-GAGCAGGCTGGAGAA-3'
miR-182-5p	5'-TGCGGTTTGGCAATGGTAGAAC-3'	5'-CCAGTGCAGGGTCCGAGGT-3'
GPC1	5'-GGACCTTGGCTCTGCCC-3'	5'-GTAAGGGCCAGGAAGAG-3'
GAPDH	5'-GAAGGTGAAGGTCGGAGTC-3'	5'-GAAGATGGTGATGGGATT-3'
U6 snRNA	5'-ATTGGAACGATACAGAGAAGAT-3'	5'-GGAACGCTTCACGAATTT-3'

© 2014 by the authors; licensee MDPI, Basel, Switzerland. This article is an open access article distributed under the terms and conditions of the Creative Commons Attribution license (http://creativecommons.org/licenses/by/3.0/).
